# The criticome as the window of becoming: Toward a novel and comprehensive framework for understanding the critical period of information integration in human development

**DOI:** 10.61373/bh026i.0021

**Published:** 2026-06-02

**Authors:** Michel Cuenod, Julio Licinio, Kim Q. Do

**Affiliations:** 1 Faculty of Biology and Medicine, Center for Psychiatric Neuroscience, Lausanne University, Lausanne, Switzerland; 2 Departments of Psychiatry & Neuroscience & Physiology, State University of New York, Upstate Medical University, Syracuse, NY 13210, USA

**Keywords:** Critical periods, criticome, neurodevelopment, parvalbumin interneurons, schizophrenia, synaptic plasticity

## Abstract

Critical periods of synaptic plasticity represent windows of extraordinary neural malleability that fundamentally shape brain architecture and function and can determine brain health for decades to come. Yet neuroscience lacks adequate terminology to describe the totality of experiential information integrated during these periods. We propose a conceptually novel term: criticome, as the complete ensemble of sensory, motor, social, cultural, and environmental information recorded during critical periods from prenatal development through approximately age 25, with the recognition that this boundary is probabilistic and domain-specific rather than fixed. Our original framework is grounded in six coupled neurobiological mechanisms: GABAergic regulation through parvalbumin-positive interneurons, perineuronal net dynamics, myelination, epigenetic regulation, neuromodulatory maturation, and developmental synaptic pruning. Their collective state determines what experience can be integrated and how stable the outcome is. The criticome reframes cultural specificity, implicit bias, aesthetic preference, and interpersonal attraction as variations in integrated content rather than differences in brain architecture, and reconceptualizes autism, schizophrenia, post-traumatic stress, major depression, and culture-bound syndromes as developmental rather than purely synaptic disorders. The same plasticity that allows Mozart to emerge from early auditory exposure also produces the lasting damage seen in institutional deprivation, ideological indoctrination, and chronic geopolitical trauma. This framework carries practical consequences for therapeutic timing, educational policy, cultural competence in mental healthcare, and interventions aimed at reopening plasticity in adulthood, while simultaneously raising urgent questions about how screen-mediated environments now shape criticomes during the windows when neural architecture is most malleable.

## Introduction: The critical period paradox

Critical periods of heightened synaptic plasticity, first described by Hubel and Wiesel in the visual system (1), represent one of neuroscience’s most profound discoveries. These temporal windows of enhanced neural malleability are now recognized across multiple brain regions and sensory modalities, each region and modality governed by specific molecular mechanisms, including parvalbumin-positive interneuron maturation (2), perineuronal net formation (3), and myelination patterns (4). They are also recognized at the behavioral level. Language acquisition and birdsong learning are the two best-studied examples: in both, a developmental window opens early, allows the rapid integration of a complex sensorimotor code from environmental exposure, and then closes, after which acquisition becomes effortful and incomplete (5, 6). The phenomenon was noted by folk observation long before it was named by neuroscience. The Brazilian proverb *papagaio velho não aprende a falar* (an old parrot does not learn to speak) captures, in five words, what the molecular work has since confirmed: there is a window for vocal learning, and it closes. Yet despite decades of research into the cellular and molecular mechanisms governing critical periods, we lack conceptual frameworks adequate to describe their ultimate product: the totality of experiential information integrated during these periods of heightened plasticity.

This conceptual gap represents more than semantic imprecision. Without appropriate terminology, we cannot adequately theorize about how early experience creates the neural substrates of consciousness, culture, and individual identity. Existing terms (memory, the unconscious, and cultural conditioning) capture fragments but miss the integrated whole. We propose that this gap can be addressed through the introduction of a novel concept: the criticome.

## Defining the criticome

The criticome encompasses the complete ensemble of experiential information recorded in neural networks during critical periods of synaptic plasticity, spanning prenatal development through approximately age 25. This includes the following elements:

## Sensory integration

All visual, auditory, olfactory, gustatory, and somatosensory experiences occurring during periods of heightened plasticity, along with their contextual associations and emotional valences.

## Motor encoding

Patterns of movement, gesture, and embodied interaction with the physical environment that become embedded in developing neural circuits.

## Social scaffolding

Interpersonal dynamics, attachment patterns, and social hierarchies that shape developing social brain networks during their periods of maximal plasticity.

## Cultural transmission

Language acquisition, symbolic systems, ritual behaviors, and cultural practices that become neurally embedded during critical developmental windows.

## Environmental context

The physical, biological, and social contexts within which development occurs, including architectural spaces, natural environments, and socioeconomic conditions.

**Figure 1.**
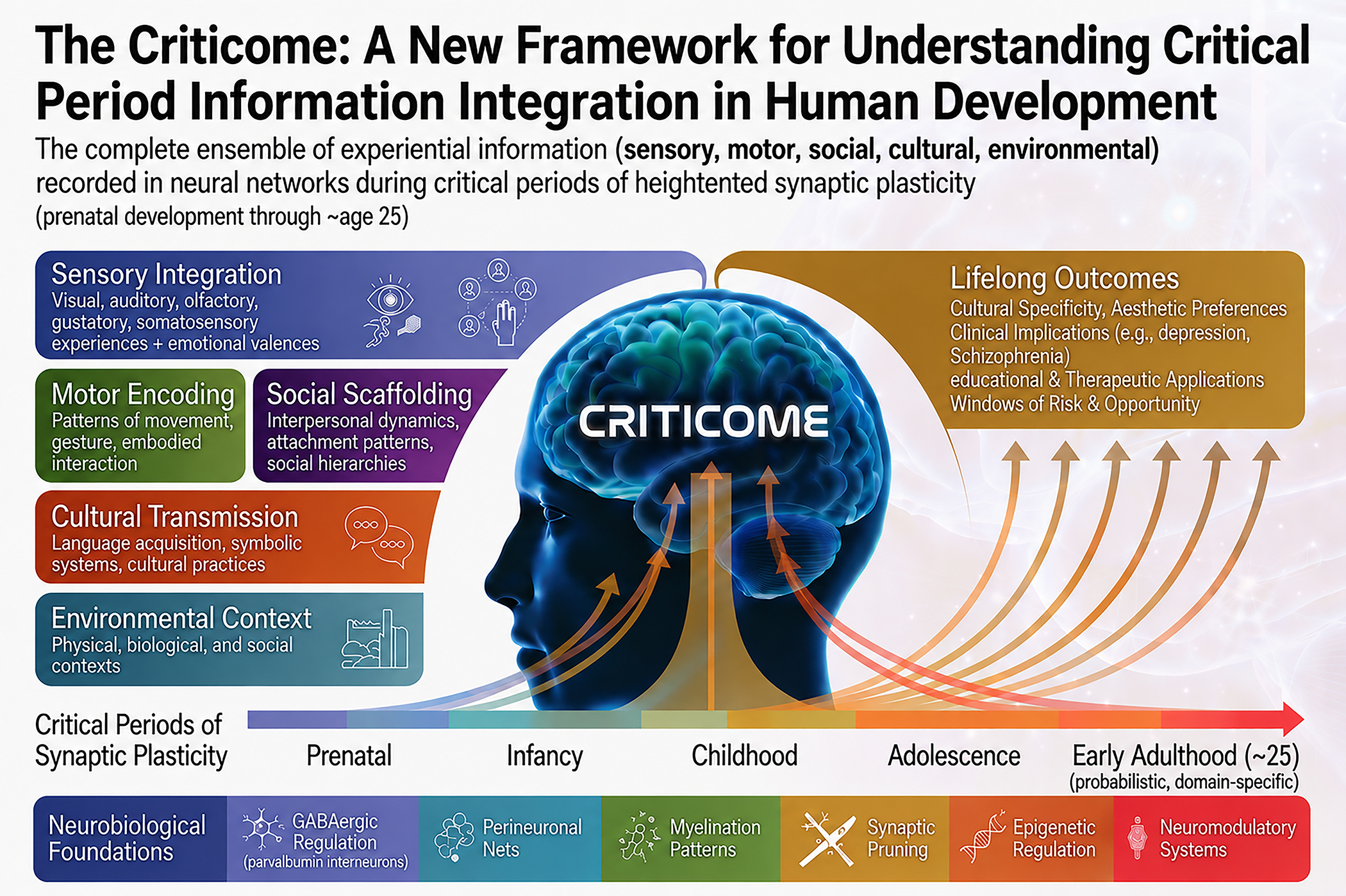
Criticome: the window of becoming. Five categories of experiential information (sensory, motor, social, cultural, and environmental) become integrated into developing neural architecture during critical periods of synaptic plasticity, spanning prenatal development through approximately age 25; the upper boundary is probabilistic and domain-specific. Six neurobiological mechanisms govern these windows: GABAergic regulation through parvalbumin-positive interneurons, perineuronal nets, myelination, synaptic pruning, epigenetic regulation, and neuromodulatory systems. Integration shapes lifelong outcomes, including cultural specificity, aesthetic preferences, clinical implications (e.g., depression and schizophrenia), educational and therapeutic applications, and windows of risk and opportunity. Created by JL with iterative assistance from Grok (xAI), Gemini (Google), and ChatGPT (OpenAI).

The criticome differs from traditional concepts of memory or conditioning in several crucial ways. First, it encompasses information integrated specifically during periods of heightened plasticity, when neural circuits are maximally malleable, and experience can fundamentally alter brain architecture. Second, it includes not just discrete memories but the entire experiential matrix that shapes developing neural networks. Third, it recognizes that this information becomes structurally embedded in the brain’s architecture in ways that may not be consciously accessible yet profoundly influence perception, cognition, and behavior throughout life. The five categories of input, the developmental windows during which they are integrated, and the lifelong domains they shape are summarized in Figure 1.

## The closure boundary: Probabilistic, domain-specific, and variable

The phrase “approximately age 25” used throughout this framework requires nuance. It is a population-level approximation, not a fixed developmental threshold. Critical period closure is probabilistic, domain-specific, and varies across individuals, biological sex, and environmental conditions. The visual cortex closes its primary window in early childhood; the auditory cortex closes for native phonemic discrimination in the first year of life; the prefrontal cortex closes far later, with synaptic pruning and myelination continuing into the third decade. Sex differences in the timing of prefrontal maturation are well documented, with female prefrontal trajectories generally consolidating earlier than male ones. Environmental adversity, including chronic stress, malnutrition, and severe early deprivation, can shift closure timing in either direction, sometimes prematurely consolidating circuits and sometimes prolonging windows of vulnerability.

The implication for research is that cohort designs should treat developmental boundaries as continuous variables rather than dichotomous cutoffs. The implication for clinical practice is that intervention windows are best understood as gradients of declining plasticity rather than hard deadlines. A 26-year-old is not categorically beyond the reach of criticome-modifying intervention; a 14-year-old is not categorically still within it for every system. The boundary the criticome framework names is real and biologically grounded, but its sharpness is more nominal than absolute, and the framework gains rather than loses by saying so.

## Neurobiological foundations

**Figure 2.**
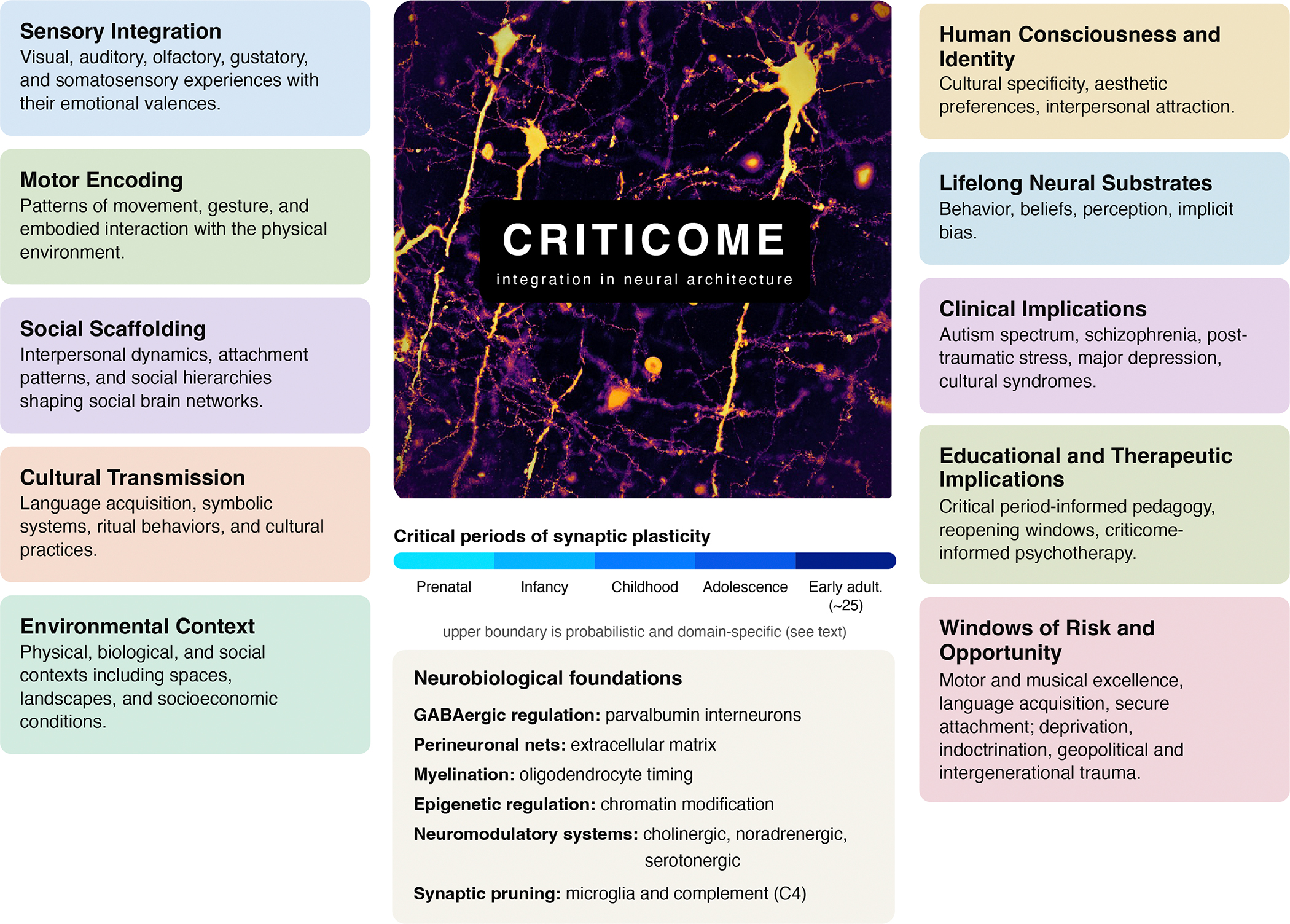
The criticome framework. Understanding the criticome. Five categories of experiential information (left: sensory integration, motor encoding, social scaffolding, cultural transmission, and environmental context) become integrated into developing neural architecture during critical periods of synaptic plasticity, spanning prenatal development through approximately age 25. The upper boundary is shown as a single label for clarity, but is in fact probabilistic and domain-specific: visual and auditory windows close in early childhood, prefrontal pruning and myelination continue into the third decade, and timing varies with individual, sex, and environmental conditions (refer to text). This integration shapes five domains of lifelong human function (right: human consciousness and identity, lifelong neural substrates, clinical implications including major depression, educational and therapeutic implications, windows of risk and opportunity). Underlying neurobiological mechanisms (GABAergic regulation, perineuronal nets, myelination, synaptic pruning, epigenetic regulation, neuromodulatory systems) are summarized in the central panel and detailed in Table 1. Created by JL with iterative assistance from Claude (Anthropic). Central image: cortical neurons (Rattus norvegicus), silver nitrate stained and pseudocolored, by Micromural123, Wikimedia Commons, licensed under CC BY 4.0.

The concept of the criticome finds strong support in contemporary neuroscience research. Critical periods are characterized by specific molecular and cellular mechanisms that create windows of enhanced synaptic plasticity. Six mechanisms are now well established: GABAergic regulation through parvalbumin-positive interneurons, extracellular matrix dynamics governed by perineuronal nets, progressive myelination of cortical circuits, experience-dependent epigenetic regulation, the neurochemical context provided by maturing neuromodulatory systems, and developmental synaptic pruning. The cellular substrates and the role each plays in critical period plasticity are summarized in Figure 2 and Table 1 (7).

Several features of these mechanisms deserve emphasis beyond what the table conveys. The maturation of parvalbumin-positive interneurons creates the balance of excitation and inhibition necessary for critical period onset, and disruption of this balance, through genetic manipulation or pharmacological intervention, can reopen or extend critical periods (15). The formation and dissolution of perineuronal nets around fast-spiking interneurons regulates critical period timing and plasticity levels, and enzymatic degradation of these structures can restore juvenile-like plasticity in adult circuits. Experience-dependent changes in gene expression during critical periods create lasting modifications to chromatin structure that may persist throughout life. These epigenetic marks may represent one mechanism by which criticome information becomes permanently encoded.

A sixth mechanism, less often listed alongside the cellular and molecular regulators above but no less consequential, is developmental synaptic pruning. From birth, synaptic networks are shaped and refined by activity and experience, with up to half of cortical synapses being eliminated and others strengthened in a protracted process that extends, in the human prefrontal cortex, into the third decade of life. Pruning is now understood to be driven in part by microglia and complement-mediated tagging of synapses for elimination, and aberrant pruning during adolescence is a leading mechanistic hypothesis for the developmental timing of schizophrenia onset (12). The criticome framework treats pruning not as a separate phenomenon from the five mechanisms above but as their orchestrated consequence: the period during which refinement is most active is itself a window during which criticome content is selected, consolidated, or lost. What is pruned cannot be recovered; what is preserved becomes load-bearing for adult function.

## The criticome and human consciousness

The criticome framework offers new perspectives on fundamental questions about human consciousness and identity. Rather than treating consciousness as an emergent property of complex neural computation, the criticome framework suggests that consciousness is fundamentally shaped by the specific experiential information integrated during critical periods. This perspective helps explain several puzzling aspects of human psychology.

## Cultural specificity

The profound differences in perception, cognition, and behavior across cultures may reflect variations in criticome content rather than fundamental differences in brain architecture. The neural circuits are similar, but the experiential information integrated during critical periods differs dramatically.

## Implicit bias

Unconscious biases and preferences may reflect criticome content that was integrated during early critical periods, when social categories and hierarchies became embedded in developing neural networks below the threshold of conscious awareness.

## Aesthetic preferences

Individual differences in aesthetic judgment, musical taste, and artistic appreciation may reflect the specific sensory and cultural information integrated during critical periods when relevant brain regions were maximally plastic.

## Interpersonal attraction

The tendency for individuals from similar cultural backgrounds to form stronger bonds, and for “third culture kids” to preferentially associate with others who share their multicultural experience, may reflect criticome compatibility rather than conscious choice (16).

## Clinical implications

The criticome framework has significant implications for understanding psychiatric and neurological conditions. Several psychiatric disorders may reflect disruptions in critical period plasticity or pathological criticome content. Five broad clinical patterns are particularly relevant: autism spectrum disorders, schizophrenia, post-traumatic stress, culture-bound syndromes, and major depressive disorder. The proposed criticome disruption associated with each, the developmental window of greatest vulnerability, and the supporting references are summarized in Table 2 (17). A pattern across these conditions is worth naming. The criticome lens reframes psychiatric pathology as developmental rather than purely synaptic. The clinical question shifts from what is broken in the adult brain to what could not be integrated, or what was integrated incorrectly, during the windows when integration was possible. The reframing carries practical consequences for research priorities and for therapeutic timing. A natural experiment from psychiatric twin research illustrates the developmental reframing for major depressive disorder. Kendler and Halberstadt conducted joint autobiographical interviews with 14 pairs of monozygotic twins reared together who were rigorously discordant for a lifetime history of major depression, and asked the pairs themselves to narrate what had pulled their lives apart (22). In most pairs, the affected twin had suffered a wounding break in an intimate relationship, sometimes by sheer chance, sometimes channeled there by a slightly more impulsive or less planful temperament that, over decades, hardened into a divergent life. The authors call this slow magnification of small early differences cumulative continuity. With genotype held constant and the rearing family shared, what separated the depressed twin from the well twin was the accreted weight of interpersonal and occupational experience integrated across the long late-adolescent and early-adult window. The criticome lens places that finding in a mechanistic frame: prefrontal critical-period closure is gradual and protracted, and the social scaffolding integrated during its trailing edge is itself load-bearing for adult mood regulation.

## Therapeutic implications

Understanding the criticome also suggests new therapeutic approaches.

## Critical period reopening

Pharmacological and behavioral interventions that can reopen critical periods (23, 24) might allow therapeutic modification of pathological criticome content. This could provide new approaches to treating conditions rooted in early experience.

## Criticome-informed psychotherapy

Therapeutic approaches that explicitly address criticome content, through techniques like psychedelic-assisted therapy, somatic approaches, or artistic expression, might be more effective than purely cognitive interventions.

## Cultural competence

Recognition that therapist and patient may have fundamentally different criticomes, based on their developmental experiences, suggests the need for new approaches to cultural competence in mental healthcare.

## Educational implications: The criticome and learning

The criticome framework has profound implications for educational practice and policy. Despite our knowledge of sensitive periods of brain development and the overwhelming evidence of the broad spectrum of damage caused by early childhood adversity, it remains an ongoing challenge to translate this science into social policies that protect children and educational approaches that optimize learning during critical windows.

## Critical period-informed pedagogy

Understanding that different brain systems have distinct critical periods suggests the need for temporally sensitive educational interventions.

Although there is clear evidence that children have the capacity to attain fluency in any language if they are exposed to it starting at birth, the teaching of second languages continues to be delayed until early adolescence, and bilingual programs for young children are insufficiently valued. The criticome framework suggests that language exposure during early critical periods becomes structurally integrated into neural architecture in ways that later exposure cannot replicate.

## Multimodal learning environments

Techniques that integrate multimodal learning experiences can facilitate deeper understanding by activating various neural pathways, nurturing retention, and application of knowledge. The criticome framework suggests that rich, multisensory educational environments during critical periods create more robust and accessible neural representations than traditional single-modality approaches.

## Neuroeducation and individual differences

Precision brain mapping, for example, has gained traction as a method that captures detailed, reliable, individual-specific functional brain properties through extensive neuroimaging data collection from each participant. Understanding individual variations in criticome content could inform personalized educational approaches that build on each student’s unique developmental history.

## Early childhood investment

The criticome framework provides biological justification for increased investment in early childhood education. While educational reforms dedicate resources to the training, recruitment, and retention of K–12 teachers, they often do not invest sufficiently in preschool teachers. If criticome formation during early critical periods fundamentally shapes learning capacity, then early interventions may yield disproportionate returns on educational investment. The economic case for this redirection has been argued for two decades and continues to find empirical support (25).

## Windows of risk and opportunity

Critical periods represent double-edged phenomena: they are simultaneously windows of extraordinary opportunity and periods of heightened vulnerability (26, 27). The criticome framework helps explain this duality by recognizing that the same neurobiological mechanisms that enable optimal learning during critical periods also create susceptibility to disruption.

## Opportunity windows

Environmental stimuli also play a crucial role in cognitive development, particularly during the sensitive periods for learning. Early childhood education, for instance, can take advantage of critical periods for brain development by providing stimulating experiences that promote cognitive and emotional growth. During critical periods, positive experiences become deeply integrated into developing neural architecture.

## Motor learning excellence

The progression from a toddler’s tentative first steps to Roger Federer’s balletic precision on Centre Court exemplifies how motor experiences during critical periods become permanently embedded in neural architecture. Early exposure to complex motor patterns, whether grasping a tennis racquet, positioning fingers on piano keys, or coordinating dance movements, during periods of heightened plasticity creates foundational neural circuits that enable later virtuosity. This explains why elite athletes typically begin training during early childhood: not merely for practice time, but because the neural architectures required for complex motor control can only be optimally established during critical windows.

## Musical genius and auditory architecture

The transformation from young Mozart composing at the keyboard to the mature creator of symphonic masterpieces reveals how musical competence emerges through criticome integration during auditory critical periods. Early exposure to harmonic relationships, rhythmic patterns, and tonal structures during periods of heightened auditory plasticity creates neural architectures that enable extraordinary creative expression. The child who hears complex musical relationships during critical periods develops neural networks capable of processing and generating sophisticated musical ideas that remain inaccessible to those without such early exposure. This neurobiological reality explains why musical prodigies invariably begin their journey during early childhood critical periods, not simply due to parental ambition, but because the neural foundations for musical excellence can only be established when auditory systems are maximally plastic.

## Spiritual and contemplative architecture

The remarkable journey of Lhamo Dhondup, recognized as the Dalai Lama at the age of 2 years and immersed in contemplative practices from early childhood, demonstrates how spiritual frameworks integrated during critical periods shape lifelong consciousness and worldview. Early exposure to meditative traditions, philosophical concepts, and contemplative practices during periods of heightened neural plasticity creates lasting alterations in attention regulation, emotional processing, and conceptual understanding. The neural architectures established through intensive contemplative training during critical periods enable forms of consciousness and wisdom that remain largely inaccessible to those who begin such practices in adulthood. This example illustrates how criticome content encompasses not just sensory and motor skills but the deepest aspects of human consciousness and spiritual development.

## Language acquisition

Children exposed to rich linguistic environments during early critical periods develop sophisticated language processing networks that persist throughout life. The ability to distinguish phonemes, acquired through exposure during the first 10 to 12 months, becomes permanently embedded in neural architecture. Japanese children can discriminate “L” and “R” sounds during their critical window, but this capacity is lost if not reinforced through experience, demonstrating how criticome formation involves both acquisition and preservation of neural capabilities.

## Musical training

Early musical exposure during auditory critical periods creates enhanced auditory processing capabilities that facilitate both musical and linguistic abilities. Children who receive musical training during critical periods develop neural networks that support superior pitch discrimination, temporal processing, and even mathematical abilities (28).

## Social-emotional learning

Secure attachment relationships formed during early critical periods create neural templates for future interpersonal relationships, establishing criticome content that influences emotional regulation and social competence throughout life.

## Risk windows

The same plasticity mechanisms that enable opportunity also create vulnerability. The sensorimotor-association axis of cortical development progresses hierarchically, with critical periods cascading from sensory to association areas, culminating in the prefrontal cortex during adolescence (29). Disruptions during these periods can have lasting effects.

## Early institutional care

Children raised in Romanian orphanages during critical periods show profound developmental delays across multiple domains. The presentation demonstrates how deprivation during critical windows creates lasting neural and behavioral consequences that persist even after environmental improvement (30, 31).

## Exploitation by totalitarian systems

The Nazi Hitlerjugend represents a dark example of how critical periods can be exploited. Young people exposed to ideological indoctrination during periods of heightened plasticity developed lasting neural architectures that supported extremist beliefs and behaviors. The progression from youth programs to SS membership illustrates how criticome content integrated during critical periods can shape lifelong worldviews and moral frameworks.

## Contemporary geopolitical trauma

Current global conflicts expose children and adolescents to violence, displacement, and trauma during critical developmental windows. Whether experiencing war directly, witnessing violence through media, or living with the chronic stress of geopolitical instability, today’s youth are having these traumatic experiences integrated into their developing criticomes. This creates lasting neural and psychological vulnerabilities that may persist throughout their lives, affecting not only individual well-being but also intergenerational patterns of trauma and social functioning.

## Intergenerational trauma

As Professor François Ansermet notes, “When a child is mistreated, traumatized, it is not only a child who is affected, it is also an entire lineage.” Maltreatment during critical periods becomes integrated into the criticome in ways that can perpetuate across generations, as individuals replicate the relational patterns they experienced during development.

## Sensory deprivation

Amblyopia occurs when the visual input to one eye is impaired during early childhood, demonstrating how critical period disruption can create permanent functional deficits (32).

## Social isolation

Reduced social interaction during social brain critical periods may impair the development of interpersonal neural networks.

## Intervention timing

Potential investigatory approaches could include transcranial magnetic stimulation to alter synaptic inhibition and excitation, dietary enrichment with iron and sphingolipids to support myelinogenesis, short-term administration of benzodiazepines or valproate to shift the timing of plastic periods, or environmental enrichment to enhance ongoing neuroplasticity. The criticome framework suggests that interventions must be timed to match critical period windows for maximum effectiveness.

## Accessing the criticome

The criticome framework helps explain why certain experiences seem to provide privileged access to early developmental content. The criticome framework also provides insight into individual differences in aesthetic experience and artistic creation. Ferdinand Hodler’s painting “Le Muveran” may resonate powerfully with individuals whose criticomes include early exposure to Alpine landscapes, while leaving others unmoved. This suggests that artistic appreciation reflects not just cultural learning but fundamental neural architectures established during critical periods.

A sentence from *Finnegans Wake* does not appear to make sense. Neither does a sentence from the letters of Lucia Joyce, James Joyce’s daughter. The surface signs are the same: ruptured syntax, coined words, associations that skip the rails of ordinary logic. Yet one writer is among the most celebrated novelists of the twentieth century, the other a patient diagnosed with schizophrenia. Why? Carl Jung, who briefly treated Lucia in 1934, gave the sharpest answer on record: father and daughter were “like two people going to the bottom of a river, one falling and the other diving” (33). The river is the same. The descent is not. The criticome offers a neurobiological reading of that asymmetry. Joyce composed from a criticome integrated under intact critical-period regulation, then chose, as an adult, to navigate its depths; Lucia’s late-adolescent prefrontal window closed on a disrupted integration, and the same depths drew her down without her control or consent.

The universal human tendency toward nostalgia finds new meaning within the criticome framework. Poetry across cultures, from Joachim du Bellay’s longing for his “petit village” to Charles Baudelaire’s “douce langue natale” to Friedrich Hölderlin’s “Heimkunft,” represents attempts to access and express criticome content. As the presentation suggests, “One of the functions of poetry would be to try to express this inexpressible.”

## Artistic expression

The universal human drive toward artistic creation, whether through visual arts, music, literature, or movement, may represent attempts to externalize and communicate criticome content. Proust’s madeleine represents just one example of how sensory experiences can trigger access to deeply embedded developmental memories.

## Psychedelic experiences

Recent research on psychedelic therapeutics suggests these substances may temporarily reactivate critical period-like plasticity (34). This could explain why psychedelic experiences often involve access to early memories and fundamental shifts in perspective. They may allow temporary access to and modification of criticome content.

## Contemplative practices

Meditation, prayer, and other contemplative practices may provide pathways for accessing criticome content through altered states of consciousness that bypass normal cognitive filters.

## Dreams

The preferential access to developmental content during dreaming may reflect the brain’s natural mechanisms for processing and integrating criticome information during sleep.

## Evolutionary perspectives

The criticome framework also offers new perspectives on human evolution and cultural transmission. Critical periods may have evolved as mechanisms for cultural learning and adaptation.

## Cultural evolution

The extended critical periods characteristic of human development may represent adaptations for cultural learning. Unlike other species, humans require extensive cultural information to survive and reproduce successfully. Extended critical periods allow for the integration of complex cultural knowledge.

## Group cohesion

Shared criticome content within cultural groups creates the psychological foundations for social cohesion and cooperation. Individuals with similar criticomes experience intuitive understanding and trust, while those with different criticomes may experience conflict or misunderstanding.

## Adaptive plasticity

Critical periods allow developmental plasticity in response to local environmental conditions. The criticome ensures that individuals develop neural architectures optimized for their specific ecological and cultural niches.

## Future directions and research agenda

The criticome framework suggests several crucial research directions.

## Measurement and assessment

Development of neuroimaging and behavioral methods for assessing criticome content and its neural substrates. This might include techniques for measuring critical period-specific neural architecture and its relationship to behavioral phenotypes.

## Cellular and molecular mechanisms

It is crucial to study at the cellular and molecular level, not only through neuroimaging, but also the spatiotemporal development of neural circuitry related to various critical phases and systems. This includes detailed investigation of excitatory/inhibitory balance, neuromodulatory systems, and epigenetic regulation with a view toward targeted prevention and therapeutics. Such research could include the study of markers of redox hub pathways (35, 36) and investigation of neuroprotective agents that might optimize critical period outcomes or ameliorate disruptions.

## Intervention studies

Research on interventions that can modify criticome content, either through critical period reopening or through experiences that access and modify embedded content.

## Cross-cultural studies

Systematic investigation of how different cultural contexts during critical periods create distinct neural architectures and behavioral patterns.

## Clinical applications

Development of criticome-informed diagnostic and therapeutic approaches for psychiatric and neurological conditions.

## Technological integration

Investigation of how digital environments and virtual reality experiences during critical periods might create novel forms of criticome content. This question has acquired particular urgency given the unprecedented exposure of contemporary children and adolescents to screen-mediated experience (37).

## Conclusion: Toward a new neuroscience of human experience

The criticome framework represents more than a new term. It offers a paradigm shift in how we understand the relationship between early experience and lifelong neural function. By recognizing that critical periods create not just memories but fundamental neural architectures that shape consciousness itself, we can begin to address some of neuroscience’s most challenging questions.

This framework suggests that individual differences in perception, cognition, and behavior may reflect variations in criticome content rather than fundamental differences in brain architecture. It offers new perspectives on cultural transmission, interpersonal relationships, and psychiatric pathology. Most importantly, it provides a neurobiologically grounded foundation for understanding how early experience creates the neural substrates of human identity and consciousness.

The implications extend far beyond neuroscience. In an increasingly globalized world, understanding how early cultural experience shapes neural architecture could inform approaches to education, mental healthcare, and cross-cultural understanding. As we develop new technologies that can influence neural plasticity, the criticome framework provides essential guidance for ensuring that interventions enhance rather than disrupt healthy development.

The journey toward understanding the criticome has only begun. But by naming and conceptualizing this fundamental aspect of human development, we take a crucial step toward a more complete neuroscience of human experience, one that honors both the biological mechanisms of neural plasticity and the profound role of cultural and environmental context in shaping who we become. It is the totality of those mechanisms, which we now refer to as the criticome, rather than a single reductionistic perspective, that makes us who we are.

## Figures and Tables

**Table 1.** Neurobiological mechanisms governing critical period plasticity.

Mechanism	Cellular substrate	Role in critical periods	Reference
GABAergic regulation	Parvalbumin-positive interneurons	Maturation creates excitation-inhibition balance necessary for critical period onset; disruption can reopen or extend critical periods	8
Extracellular matrix dynamics	Perineuronal nets surrounding fast-spiking interneurons	Formation regulates critical period timing and plasticity levels; enzymatic degradation restores juvenile-like plasticity in adult circuits	9
Myelination	Oligodendrocytes and glial regulators	Progressive myelination of cortical circuits both enables and constrains plasticity; regional timing creates distinct critical periods for different functions	10, 11
Synaptic pruning	Microglia and complement-mediated synaptic tagging	Activity-dependent elimination of excess synapses refines neural circuits throughout childhood and adolescence; aberrant pruning is a leading mechanistic hypothesis for the developmental timing of schizophrenia	12
Epigenetic regulation	Chromatin structure	Experience-dependent gene expression changes create lasting modifications that may persist throughout life; one mechanism by which criticome information becomes permanently encoded	13
Neuromodulatory systems	Cholinergic, noradrenergic, serotonergic networks	Provides neurochemical context for plasticity; developmental disruption produces lasting effects on brain function and behavior	14

**Table 2.** Clinical conditions associated with criticome disruption.

Condition	Proposed criticome disruption	Developmental window	Reference
Autism spectrum disorders	Altered critical period timing and duration; experiential information integrated differently, producing distinct neural architectures and behavioral phenotypes	Early childhood across multiple sensory and association systems	17
Schizophrenia	Disrupted parvalbumin-positive interneuron maturation and critical period regulation in prefrontal cortex; impaired criticome integration during late adolescent window	Late adolescence (prefrontal cortex closure)	18, 19
Post-traumatic stress	Trauma integrated into criticome in ways that fundamentally alter brain architecture and stress responsivity	Throughout childhood and adolescence; effects most profound when occurring in early critical windows	20
Cultural syndromes	Cultural beliefs and practices embedded in neural circuits during critical periods, predisposing to particular symptom patterns	Variable, dependent on cultural exposures during childhood and adolescence	21
Major depressive disorder	Cumulative continuity of adverse interpersonal and occupational experience integrated during the protracted prefrontal critical-period window; small temperamental differences channel divergent life experience that magnifies across decades	Late adolescence through mid-twenties (prefrontal window); cumulative across the lifespan	22
